# Identification and functional analysis of gene cluster involvement in biosynthesis of the cyclic lipopeptide antibiotic pelgipeptin produced by *Paenibacillus elgii*

**DOI:** 10.1186/1471-2180-12-197

**Published:** 2012-09-08

**Authors:** Chao-Dong Qian, Tian-Zhe Liu, Shuang-Lin Zhou, Rui Ding, Wen-Peng Zhao, Ou Li, Xue-Chang Wu

**Affiliations:** 1 Institute of Microbiology, College of Life Sciences, Zhejiang University, Hangzhou, Zhejiang Province, P.R.China; 2 Zhejiang Pharmaceutical College, Ningbo, Zhejiang Province, P.R.China

**Keywords:** Non-ribosomal peptide, Biosynthesis, Gene cluster, Antimicrobial agent

## Abstract

**Background:**

Pelgipeptin, a potent antibacterial and antifungal agent, is a non-ribosomally synthesised lipopeptide antibiotic. This compound consists of a β-hydroxy fatty acid and nine amino acids. To date, there is no information about its biosynthetic pathway.

**Results:**

A potential pelgipeptin synthetase gene cluster (*plp*) was identified from *Paenibacillus elgii* B69 through genome analysis. The gene cluster spans 40.8 kb with eight open reading frames. Among the genes in this cluster, three large genes, *plpD*, *plpE*, and *plpF,* were shown to encode non-ribosomal peptide synthetases (NRPSs), with one, seven, and one module(s), respectively. Bioinformatic analysis of the substrate specificity of all nine adenylation domains indicated that the sequence of the NRPS modules is well collinear with the order of amino acids in pelgipeptin. Additional biochemical analysis of four recombinant adenylation domains (PlpD A1, PlpE A1, PlpE A3, and PlpF A1) provided further evidence that the *plp* gene cluster involved in pelgipeptin biosynthesis.

**Conclusions:**

In this study, a gene cluster (*plp*) responsible for the biosynthesis of pelgipeptin was identified from the genome sequence of *Paenibacillus elgii* B69. The identification of the *plp* gene cluster provides an opportunity to develop novel lipopeptide antibiotics by genetic engineering.

## Background

**Figure 1:**
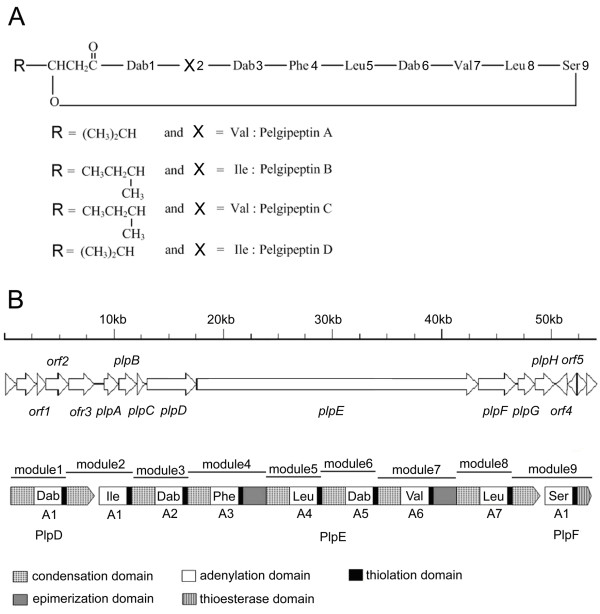
**Pelgipeptin and the genes responsible for its biosynthesis.** (**A**) Primary structure of pelgipeptin. (**B**) The *plp* gene cluster and domain organisation of the NRPS.

The intensive use of chemical pesticides to treat plant diseases has resulted in various problems such as severe environmental pollution, food safety concerns, and emergence of drug resistance. Biological control using microorganisms or their metabolites, a more rational and safer method, has emerged as a promising alternative to suppress plant pathogens and reduce the use of agrochemicals [1,2]. Pelgipeptins, a group of natural active compounds isolated from *Paenibacillus elgii* B69, are potential biological control agents [1]. This group of antibiotics has a general structure composed of a cyclic nonapeptide moiety and a β-hydroxy fatty acid. Four analogues of pelgipeptin have been identified and characterised [3]. These analogues are highly similar in structure and differ only in one amino acid unit or in the lipid acid (Figure1A). Pelgipeptin exhibits broad-spectrum antimicrobial activity against pathogenic bacteria and fungi, including *Staphylococcus aureusEnterococcus faecalisEscherichia coliCandida albicansFusarium oxysporumF. graminearumF. moniliformeRhizoctonia solani*, and *Colletotrichum lini*[1,3]. This compound effectively inhibited the development of sheath blight caused by *R. solani* on rice in a preliminary evaluation of the *in vivo* efficacy of pelgipeptin [1].

Similar to polymyxin and fusaricidin from *P. polymyxa,* pelgipeptin, containing non-proteinogenic and D-amino acids, must be synthesised by a non-ribosomal peptide synthetase (NRPS). NRPS is a large multifunctional enzyme that has modular structures [4]. Each NRPS module catalyses the incorporation of a specific substrate into the growing product. A typical module consists of three enzymatic domains, namely, adenylation (A), thiolation (T; also known as peptidyl carrier protein), and condensation (C) domains. The A domain selects and activates a specific amino acid substrate, the T domain is responsible for tethering the activated substrate to the 4′-phosphopanthetheinyl cofactor, and the C domain catalyses peptide bond formation between the elongating peptide and a new amino acid. In addition to these core domains, the terminal thioesterase (TE) and epimerisation (E) domains, as well as several other tailoring domains, may also be present in NRPS modules. The order of modules of an NRPS is, in many cases, collinear to the amino acid sequence of the corresponding peptide product. The collinearity rule of NRPS systems combined with knowledge of the specificity-conferring code of A domain allow for the prediction and amino acid modification of peptide fragments synthesised by corresponding NRPS [5]. However, few NRPS sequences have been extensively described in comparison with the number of known peptide products, limiting the study of the principles of non-ribosomal peptide synthesis and the development of new bioactive peptides by genetic engineering. In this study, we identified and analysed a gene cluster involved in the biosynthesis of pelgipeptin and provided biochemical data for the collinearity of this peptide assembly line.

## Methods

### Bacteria strains and culture conditions

*P. elgii* B69, isolated from a soil sample [1], was cultured in nutrient broth. *E. coli* DH5α, for gene manipulation, and *E. coli* BL21 (DE3), for overexpression of recombinant proteins, were cultivated on Luria-Bertani medium.

### Identification and in silico analysis of *plp* gene cluster in *P. elgii* B69

The draft genome sequence of the strain was used to build a database in Bioedit to identify the putative NRPS genes in *P. elgii* B69 (http://www.mbio.ncsu.edu/BioEdit/bioedit.html). The first and second C domains of PmxE (GenBank EU371992), which is a polymyxin synthetase subunit, were compared with the created database using local BLAST searches [6] as implemented in Bioedit. Amino acid sequence homology searches were performed using the BLAST server at the National Centre for Biotechnology Information (NCBI, http://www.ncbi.nlm.nih.gov/BLAST/) site. NRPS domains were identified by PKS/NRPS analysis (http://nrps.igs.umaryland.edu/nrps/) [7]. Prediction of 10 amino acids located at the substrate-binding pocket of the A domain and substrate specificity prediction were performed using the web-based program NRPS predictor (http://ab.inf.uni-tuebingen.de/software/NRPSpredictor/) [8].

### Cloning, expression and purification of A domains

We synthesised four sets of specific forward and reverse primers: plpD-A1-F (CTAGCCATGGAAAACATTTTGACCCG) and plpD-A1-R (CACCTCGAGTTCGTACTCCGCTCCG); plpE-A1-F (GACACCATGGATTTGTTGTCGGAAG) and plpE-A1-R (ATCCTCGAGCACGAACTCCACGCCGGTT); and plpE-A3-F (CTAGCCATGGCGGCGGAGCAGACAC) and plpE-A3-R (CCCAAGCTTCGCGACGTAGTCGGCTC); and plpF-A1-F (CTAGCTAGCTTGTCCGACTCCGAG) and plpF-A1-R (GCGGATCCTCACTCCAGTCCGGTCT) to amplify the A domains of PlpD A1, PlpE A1, PlpE A3, and PlpF A1. The genes encoding these A domains were PCR-amplified from the genomic DNA of *P. elgii* B69 and cloned into pET28a vector. The recombinant plasmid was transformed into *E. coli* DH5α for gene manipulation. After transformation into *E. coli* BL21 (DE3), the recombinant proteins were overexpressed and produced as described previously [9].

BL21 strains expressing each A domain were grown in Luria–Bertani medium supplemented with 50 μg/ml kanamycin at 37 °C until its OD_600_ reached about 0.5. Gene expression was induced by 0.1 mM isopropyl-b-D-thiogalactopyranoside at 30 °C for 4 h. Cells were harvested by centrifugation, resuspended in buffer A (40 mM Tris–HCl, 200 mM NaCl, 20 mM imidazole, pH 8.0), and lysed by sonication on ice. The lysates were centrifuged at 12 000 g for 30 min at 4 °C, and the supernatants were loaded onto a Ni Sepharose 6 FF (GE Healthcare) column. The column was washed with five bed volumes of buffer A, followed by five bed volumes of buffer B (40 mM Tris–HCl, 200 mM NaCl, 60 mM imidazole, pH 8.0). The recombinant proteins were then eluted by buffer C (40 mM Tris–HCl, 200 mM NaCl, 150 mM imidazole, pH 8.0). Purified proteins were detected by 10 % sodium dodecyl sulphate-polyacrylamide gel electrophoresis (SDS-PAGE) and dialysed against buffer D (40 mM Tris–HCl, pH 8.0, 200 mM NaCl, and 1 mM dithiothreitol). Protein concentration was determined by the bicinchoninic acid protein assay (Pierce, USA) using bovine serum albumin as the standard.

### Determination of substrate specificity

The substrate selectivity of each of the A domains was determined using a non-radioactive assay [10]. The reaction mixture (40 μl) contained 0.5 μM recombinant A domain, 0.2 U/ml inorganic pyrophosphatase, 5 mM ATP, 100 mM NaCl, 10 mM MgCl_2_, and 6 mM amino acid in 50 mM Tris–HCl (pH 7.5). Reactions were started by the addition of ATP and incubated at 25 °C. The reactions were terminated by the addition of molybdate/malachite green reagent. After 15 min of colour development, optical density was measured at 600 nm on a microplate reader (Multiscan MK3, Thermo Electron Co. Ltd., Shanghai, China). A reaction mixture lacking the recombinant A domain was used as a negative control.

### Nucleotide sequence accession numbers

The DNA sequences for the pelgipeptin biosynthetic gene cluster in *P. elgii* B69 was deposited in the GenBank under accession number JQ745271.

## Results and discussion

### Analysis of the organisation of the *plp* gene cluster and its flanking regions

We recently completed the draft genome sequence of the pelgipeptin-producing bacterium *P. elgii* B69, in which at least 5 NRPS-related biosynthetic gene clusters were found within its 7,981,270 bp long scaffold [11]. Further inspection revealed that several NRPS genes located in scaffolds 3 and 43 were probably related with pelgipeptin biosynthesis. The gaps between and within these two scaffolds were filled by sequencing PCR products. These efforts resulted in a complete NRPS gene cluster (*plp*), harbouring eight open reading frames (ORFs), which could be assigned to pelgipeptin biosynthesis. These ORFs (designated *plpA-plpH*) were transcribed in the same direction (Figure1B). Upstream of the *plp* locus, two genes (ORF2 and ORF3) encoding proteins with similarities to heparinase II/III family proteins (YP_003243728 and YP_003243727, respectively) were transcribed in the same direction and were considered not to be involved in pelgipeptin production. Further upstream, a third ORF (ORF1), with TGA stop codon within ORF2, was found to encode a protein with high similarity to short-chain dehydrogenases/reductases (ZP_08509633) and was also considered not involved in the pelgipeptin biosynthesis. Downstream of the *plpF* gene, four genes encoding putative ABC transporter proteins were found. PlpG and PlpH, shared 72% and 69% identities with PmxC and PmxD, respectively, which were considered responsible for the secretion of polymyxin produced by *P. polymyxa*[12]. This transport activity may be needed for the transport of pelgipeptin out of the cell, and therefore, the gene products were attributed to pelgipeptin biosynthesis. The other two genes (ORF4 and ORF5) encoding putative nitrate/sulphonate/bicarbonate ABC transporter proteins were transcribed in the opposite direction and were considered less likely to be involved in pelgipeptin production, although further evidence will be required before this can be decided unequivocally. The putative ORFs and the genetic organisation of the chromosomal region containing these sequences are depicted in Figure1B.

### Genes encoding NRPS

As shown in Figure1B, three NRPS genes, *plpDplpE*, and *plpF*, are present in the *plp* cluster, and these genes encode proteins with estimated molecular masses of 171.8, 951.3, and 122.9 kDa, respectively. The modules and domains of pelgipeptin synthetase were analysed as described in the “Materials and methods” section above. PlpD, containing four domains (C-A-T-C) (Figure1B), had an N-terminal C domain, which shared 43% identity with the starter C domain of PmxE [12]. The amino acid predicted specific for the A domain of PlpD was 2,4-diaminobutyric acid (Dab) (Table1). The presence of a starter C domain in PlpD, and the specificity of the module for Dab are both consistent with this module providing the first amino acid of the pelgipeptin peptide, and therefore the fatty acid side chain should be connected to the peptide at this residue [13]. PlpE, containing 23 domains (A-T-C-A-T-C-A-T-E-C-A-T-C-A-T-C-A-T-E-C-A-T-C), comprises seven modules and a C-domain. The substrate specificities of the seven PlpE A domains were predicted to activate the amino acids Ile, Dab, Phe, Leu, Dab, Val, and Leu, respectively. Two modules contain an epimerisation domain, indicating that the related activated amino acids (Phe and Val) may be converted into the D-configuration. Three domains (A-T-TE) were present in PlpF, and the predicted amino acid specific for the A domain was Ser. The last domain of this megasynthase was a thioesterase domain, indicating that PlpF may be required for the release and cyclisation of the synthesised lipopeptides. These results indicate that *plpD* is the first and *plpF* the last gene involved in pelgipeptin biosynthesis. Thus, the number of A domains, order of modules for amino acid assembly, and location of epimerisation domains perfectly correspond to the structural characteristics of pelgipeptin (Figure1), suggesting that the *plp* gene cluster may be responsible for the synthesis of pelgipeptin in the B69 strain. 

**Table 1:** Predicted amino acids of adenylation domains in the Plp synthetase

**A-domain**	**Amino acid at PheA residue**^**a**^										**Predicted substrate**
	**235**	**236**	**239**	**278**	**299**	**301**	**322**	**330**	**331**	**517**	
PlpD A1	D	V	G	E	I	S	A	I	D	K	Dab
PlpE A1	D	G	F	F	L	G	V	V	F	K	Ile
PlpE A2	D	V	G	E	I	S	A	I	D	K	Dab
PlpE A3	D	A	W	T	I	A	A	I	C	K	Phe
PlpE A4	D	A	W	I	I	G	A	I	V	K	Leu
PlpE A5	D	V	G	E	I	S	A	I	D	K	Dab
PlpE A6	D	A	F	W	I	G	G	T	F	K	Val
PlpE A7	D	A	W	I	I	G	A	I	V	K	Leu
PlpF A1	D	V	W	H	F	S	L	V	D	K	Ser

^a^The residues were numbered according to the corresponding residues of PheA.

### In vitro assay of adenylation domains

**Figure 2:**
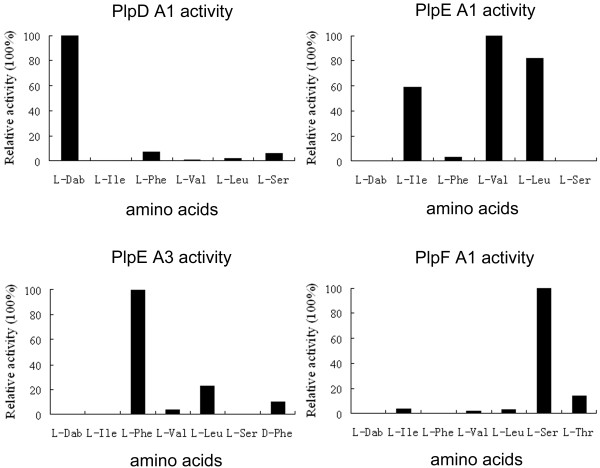
**Substrate specificity of the A domains by non-radioactive assay.** The assay was performed with 20 different proteogenic amino acids plus L-Dab and D-Phe. The highest activity was set at 100%. Only amino acids related to the composition of pelgipeptin are shown. Other amino acids with relative activities < 5% are not shown.

The substrate specificity of four A domains, PlpD A1, PlpE A1, PlpE A3, and PlpF A1 were determined through a non-radioactive assay to link further the *plp* gene cluster to pelgipeptin synthesis. The reason for our selection of PlpD A1, PlpE A3, and PlpF A1 was that their predicted products (Dab, Phe, and Ser, respectively) were characteristic amino acids of pelgipeptin. The predicted product of Plp E A1 was Ile, but the corresponding amino acid (position 2) in pelgipeptin was variable (Ile or Val). This is the reason for our selection of PlpE A1. Recombinant A-domain proteins were expressed and purified as described in the “Materials and methods” section above. All proteins with satisfactory yield (about 10 mg/L of culture) and purity (>95%) were obtained in soluble form. The substrate selectivity of A domains was determined with the 20 proteinogenic amino acids plus L-Dab and D-Phe (Figure2). PlpD A1, PlpE A3, and Plp F A1 clearly exhibited the highest activity for L-Dab, L-Phe, and L-Ser, respectively. PlpE A1 protein, however, was found to activate L-Val (100%), L-Leu (82%), and L-Ile (52%, the highest activity was set at 100%; background was usually below 5%). Val or Ile is found in different analogues of pelgipeptins at position 2 (Figure1A), whereas no analogue with Leu at this position was detected. This phenomena may be explained by the effect of the C domain of module 2 (Figure1), because in some cases, C domains also play an important role in substrate selectivity [4,14]. In general, the four tested recombinant A domains were found to activate selectively predicted amino acids, experimentally confirming the speculation that the *plp* gene cluster involved in pelgipeptin biosynthesis.

### The *plpA* gene responsible for L-2,4-diaminobutyrate biosynthesis

The peptide core of pelgipeptin contains three non-proteinogenic amino acid L-2,4-diaminobutyrate at positions 1, 3, and 6. Several studies have indicated that this unusual amino acid is formed from aspartate β-semialdehyde catalysed by the enzyme diaminobutyrate-2-oxoglutarate transaminase [15,16]. The *plpA* gene encoded a putative homologue of this enzyme and was proposed to be responsible for L-2,4-diaminobutyrate biosynthesis in *P. elgii* B69. The deduced amino acid sequence of the *plpA* gene product (PlpA, 428 amino acids) showed 50% and 38% identity with EctB from *Halobacillus dabanensis*[15] and PvdH from *Pseudomonas aeruginosa*[16], respectively. It has been demonstrated that an important substrate of diaminobutyrate-2-oxoglutarate transaminase was aspartate β-semialdehyde, which was formed from aspartyl phosphate catalysed by aspartate-semialdehyde dehydrogenase [16]. Aspartate β-semialdehyde is also a metabolic precursor for several other amino acids, including lysine, threonine, isoleucine, methionine, and diaminopimelate. Therefore, the addition of these amino acids to the culture may be favourable to the strain for the synthesis of pelgipeptin, although most of these amino acids are not components of this lipopeptide antibiotic. This hypothesis is supported by a finding that the supplementation of a fermentation medium with amino acids listed above increased the production of pelgipeptin [3].

### The *plpB* gene encoded a predicted extracellular lipolytic enzyme

The deduced product of *plpB* gene was a putative lipase/esterase with a typical secretory signal peptide, containing three distinct domains, namely, an N domain with two positively charged lysine, a hydrophobic core domain (H domain), and a C domain with the consensus sequence A-X-A at positions 23 to 25, which was a type I SPase cleavage site [17]. Cleavage at this site would give rise to a predicted mature protein (PlpB) with 495 amino acids and a molecular mass of 53.8 kDa. A comparison of the deduced amino acid sequence of PlpB with the sequence of lipase/esterase in the EMBL and SwissProt databases showed significant homology to the nucleophilic serine region of lipase/esterase, with 36% identity to LipB from *Bacillus subtilis*[18,19]. LipB preferentially hydrolysed carboxyl esters of fatty acids with short chain lengths (less than 10 carbon atoms), indicating that it was an esterase rather than a lipase. Similar to the extracellular lipolytic enzymes from the related genus *Bacillus*, Ala replaces the first Gly of the conserved Gly-X-Ser-X-Gly pentapeptide motif in PlpB [20]. Previous studies have reported that supplementing the fermentation medium with fatty acids of various chain lengths enhanced the biosynthesis of lipopeptides containing specific fatty acid side chains [21,22]. Thus, we speculated that the predicted extracellular lipase, PlpB, may facilitate the production of pelgipeptin through hydrolysis of water-soluble carboxyl esters in cultures of strain B69.

### The *plpC* gene encoded a predicted phosphopantetheinyl transferase

The T domains of the PlpD-F must be converted from their inactive apo forms to cofactor-bearing holo forms by a specific phosphopantetheinyl transferase via phosphopantetheinylation of thiotemplates. The product of the *plpC* gene might be responsible for this conversion. The deduced protein (244 amino acids) encoded by *plpC* showed high similarity to Sfp from *B. subtilis* (38% identity, 58% similarity), Gsp from *B. brevis* (37% identity, 54% similarity), Psf-1 from *B. pumilus* (35% identity, 55% similarity), and other phosphopantetheinyl transferases associated with non-ribosomal peptide synthetases. Further analysis indicated that PlpC fell within the W/KEA subfamily of Sfp-like phosphopantetheinyl transferases, which is involved in many kinds of secondary metabolite synthesis [23].

### The N-terminal C domain

The *plp* gene cluster contained a special C domain at the N terminus of PlpD (first C domain), in addition to eight typical C domains that presumably catalyzed peptide-bond formation between the adjacent amino acid residues of pelgipeptin. Sequence alignments shown that this first C domain of PlpD had only 19-25% identity with the remaining eight C domains of PlpD, -E, and –F, but shared 31-43% identity with other first C domains of lipopeptide synthetases, such as NRPSs of surfactin [24], lichenysin [25], fengycin [26], fusaricidin [27] and polymyxin [12]. In the initiation reaction of the biosynthesis of surfactin, module 1 of SrfA alone was sufficient to catalyze the transfer of β-hydroxymyristoyl group to SrfA followed by formation of β-hydroxymyristoyl-glutamate [28]. The recent study of Choi’ group also suggested that only the N-terminal C domain of PmxE was necessary for the fatty acyl tailing of polymyxin [12]. Thus, in the initial step of pelgipeptin biosynthesis, the PlpD N-terminal C domain was proposed to catalyze the condensation of the first amino acid (Dab) with a β-hydroxy fatty acid transferred from coenzyme A.

## Conclusions

In the present study, we identified a potential pelgipeptin synthetase gene cluster (*plp*) in *P. elgii* B69 through genome analysis. The cluster spans 40.8 kb with three NRPS genes (*plpD*, *plpE*, and *plpF*). The determination of substrate specificity of four A domains, PlpD A1, PlpE A1, PlpE A3, and Plp F A1 further linked the *plp* gene cluster to pelgipeptin synthesis. We failed to provide a final proof, which could have been obtained by constructing a pelgipeptin-deficient mutant, after numerous attempts because this strain was hardly amicable to genetic manipulation. However, all the results mentioned above well supported the assignment of the *plp* gene cluster as the one responsible for the production of pelgipeptin. Our results enrich the understanding of the enzymatic action in lipopeptide biosynthesis and provide insight into the mechanism of natural product diversity.

## Competing interests

The authors declare that they have no competing interests.

## Authors’ contributions

CDQ was responsible for designing the study, bioinformatic analysis, and writing the manuscript. CDQ and TZL performed the recombinant protein preparation and biochemical experiments. SLZ made substantial contributions to data analyses and interpretation. WPZ, RD, and OL helped to revise the manuscript. XCW was responsible for the integrity of the work as a whole. All authors read and approved the final manuscript.
